# On programmed ribosomal frameshifting: the alternative proteomes

**DOI:** 10.3389/fgene.2012.00242

**Published:** 2012-11-19

**Authors:** Robin Ketteler

**Affiliations:** MRC Laboratory for Molecular Cell Biology, Translational Research Resource Centre, University College LondonLondon, UK

**Keywords:** frameshift, genomic, proteomic, screen, systems biology, high-throughput

## Abstract

Frameshifting results from two main mechanisms: genomic insertions or deletions (indels) or programmed ribosomal frameshifting. Whereas indels can disrupt normal protein function, programmed ribosomal frameshifting can result in dual-coding genes, each of which can produce multiple functional products. Here, I summarize technical advances that have made it possible to identify programmed ribosomal frameshifting events in a systematic way. The results of these studies suggest that such frameshifting occurs in all genomes, and I will discuss methods that could help characterize the resulting alternative proteomes.

## Introduction

Frameshifting is a process whereby the ribosome is guided toward a triplet nucleotide that is either shifted one nucleotide position upstream (+1 frameshift) or one nucleotide position downstream (−1 frameshift). Such frameshifting occurs in all known organisms, from *E. coli* to mammals (Namy et al., 2004; Dinman, 2012a).

**Figure 1:**
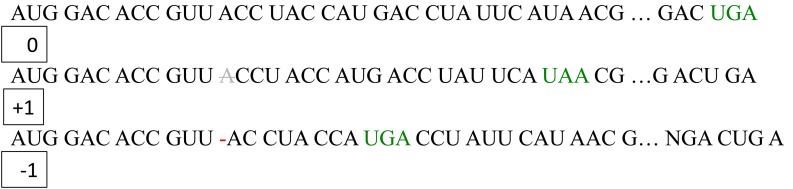
**Principle of frameshifting– +1 vs. −1.** Frameshifting can either result in skipping of one nucleotide in the mRNA resulting in +1 frameshifting or slipping back one nucleotide resulting in −1 frameshifting.

There are two main mechanisms that produce out of frame peptides: changes in the genome sequence that result in insertions or deletions (indels) and programmed ribosomal frameshifting as a consequence of the ribosome either slipping back one nucleotide (−1 frameshifting) or skipping one nucleotide (+1 frameshifting) (Figure 1). Indels generally produce non-functional proteins and are associated with either spontaneous mutations across the genome or somatic genomic instability, for instance, as a consequence of tumour progression. By contrast, programmed ribosomal frameshifting can result in dual-coding genes that produce alternative functional proteins, which form an integral part of the organism's physiology.

Programmed ribosomal frameshifting has been historically associated with viruses and retrotransposons. Retroviruses require frameshifting for replication and infection (Maia et al., 1996; Brierley and Dos Ramos, 2006; Dulude et al., 2006). For example, the HIV1 polyprotein gag-pol requires efficient −1 frameshifting for expression of the individual gag and pol gene products. This form of frameshifting usually depends on a combination of a “slippery sequence,” a spacer sequence of 1–15 nucleotides, and a stem-loop secondary RNA structure such as a pseudoknot (Figure 1) (Namy et al., 2006). The slippery sequence is generally of the type X XXY YYZ, where X denotes any nucleotide, Y denotes A or U, and Z is A, U, or C. Pseudoknots are secondary RNA substructures that contain two or more stem-loop motifs with intercalated stems. The pseudoknot or stem-loop structure in the mRNA is thought to result in pausing of the ribosome, resulting in eventual frameshifting (Namy et al., 2006). Structural evidence for this mechanism comes from the crystal structure of the mouse mammary tumor virus (MMTV) pseudoknot, which has an unpaired adenine that acts as a hinge to mediate frameshifting (Chen et al., 1996). Generally, there is a correlation between the mechanical strength of an mRNA pseudoknot and its frameshifting efficiency (Hansen et al., 2007): the stronger the pseudoknot the higher the frameshifting efficiency, although very strong pseudoknots can cause a road block that limits translation downstream (Tholstrup et al., 2011).

Other examples of mammalian genes that utilize −1 frameshifting are the mouse embryonic carcinoma differentiation regulated (EDR) gene and its human ortholog PEG10. A slippery sequence of G GGA AAC, in combination with a pseudoknot, mediates highly efficient −1 frameshifting, similar to viral frameshifting motifs (Clark et al., 2007). Recently, a programmed ribosomal −1 frameshift has been identified in the adenomatous polyposis coli (APC) mRNA in *Caenorhabditis elegans* that is mediated by a slippery sequence A AAA AAA or A AAA AAC (Baranov et al., 2011). The functional relevance of this frameshift is uncertain.

**Figure 2:**
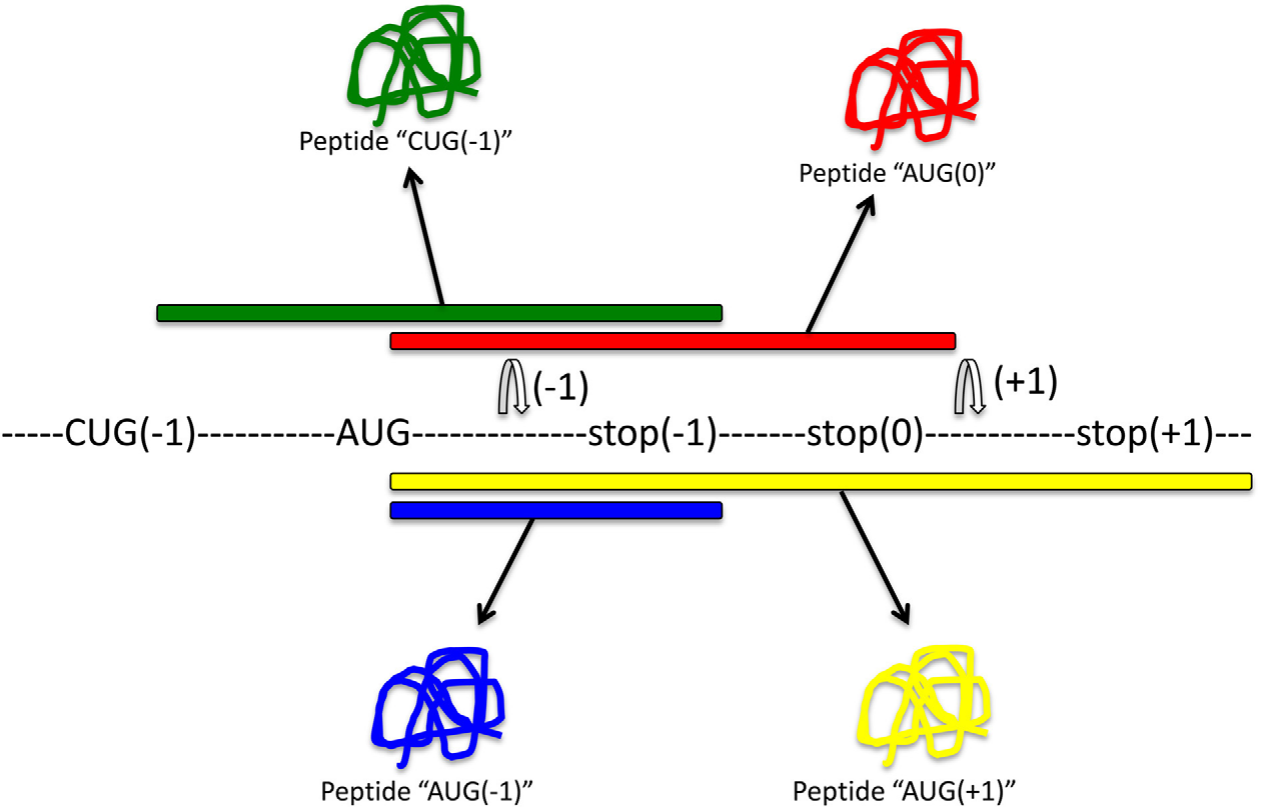
**Main sources for out-of-frame peptides.** Regular expression of this mRNA with translation initiating tRNAmet will result in expression of peptide “AUG(0)” (red). Out-of-frame peptides could arise from alternative out-of-frame CUG or AUG start sites resulting in translation of peptide “CUG(−1)” (green). Alternatively, −1 or +1 frameshift signals within the original reading frame could result in expression of out-of-frame peptides “AUG(−1)” (blue) or “AUG(+1)” (yellow), respectively.

Although the slippery sequence and pseudoknot are the most common motifs for frameshifting identified thus far, there are alternative mechanisms that may result in the production of out-of-frame proteins. Alternative splicing may contribute to frameshifting (Hiller et al., 2005), as can codon bias. For instance, rare tRNA codons can favor −1 and +1 frameshifting (Gurvich et al., 2005; Laine et al., 2008), and rare arginine codons prime mitochondrial sequences for frameshifting (Temperley et al., 2010). Moreover, CAG repeats are prone to frameshifting, which results in poly-alanine proteins that may contribute to the pathogenesis of neurodegenerative diseases (Toulouse et al., 2005). The use of the peptidyltransferase inhibitor anisomycin reduces −1 frameshifting in these cases and reduces the toxicity associated with the expanded triplet repeats. Importantly, out-of-frame proteins (compared to the standard ORFeome annotation) can also result from alternative AUG or CUG start sites (Ingolia et al., 2011), thereby considerably increasing the size of an alternative “frameshifted” proteome. Main sources of out-of-frame peptides and proteins are shown in Figure 2.

An interesting example of programmed ribosomal +1 frameshifting is that of the *ornithine decarboxylase ODC*) gene that produces the antizyme from frameshifting of the mRNA sequence (Bekaert et al., 2008). ODC catalyzes the production of polyamines, such as putrescine, spermidine, and spermine from ornithine through decarboxylation. ODC activity is terminated by the antizyme (Murakami et al., 1992)—providing an elegant mechanism for shutting down the activity of an enzyme by producing an out-of-frame antizyme from the same mRNA. This frameshifting is tightly regulated and can be enhanced by treatment of cells with polyamines (Nilsson et al., 1997). The ODC antizyme mechanism is highly conserved throughout all eukaryotes (Ivanov et al., 2000b). The frameshifting requires the UGA stop codon and a 3′ stem loop that forms a RNA pseudoknot. These RNA hallmarks are still the standard way to identify other +1 frameshifted proteins.

It has been proposed that frameshifting is a common mechanism to increase protein-coding capacity of small genomes such as those of viruses and mitochondria. In agreement with this proposal, frameshifting is common in mitochondrial genes, and genome size seems to correlate with the abundance of frameshifting (Seligmann, 2010). In fact, some organisms display a high complexity in frameshifting: In the dinoflagellate *Perkinsus marinus*, the mitochondrial gene that encodes cytochrome c oxidase subunit 1 can shift up to 10 times within the same mRNA sequence in order to produce the correct gene product (Masuda et al., 2010). Clearly, such complex frameshifting requires efficient regulatory control.

## Regulation of frameshifting

There is increasing evidence that ribosomal frameshifting is a regulated event. Several enzymatic and non-enzymatic mechanisms have been proposed that result in an enhancement of frameshifting. For instance, the production of antizyme by +1 frameshifting is enhanced by the end-products of ODC—spermine, putrescine, and spermidine (Ivanov et al., 2000a). How polyamines regulate +1 frameshifting is not well understood, but one hypothesis is that polyamine binding to RNA may enable read-through of the termination codon. Similar to polyamines, amino-glycosides such as gentamicin allow read-through of stop-codons (Martin et al., 1989; Malik et al., 2010).

It is not clear at present whether there are normal regulator proteins that enhance frameshifting efficiencies. Because −1 frameshifting is essential for retroviral gene expression, it has been proposed that chemical interference with frameshifting would be a good anti-viral strategy. Recently, a genome-wide screen to identify regulators of HIV-1 frameshifting has identified *eRF1* as an essential host gene required for −1 frameshifting (Kobayashi et al., 2010), suggesting that eRF1 may be a good therapeutic target in AIDS (Brakier-Gingras et al., 2012). In addition, it has been proposed that HIV frameshifting can be modulated by protein kinase R, as well as by factors that modulate translation efficiency such as rapamycin (Gendron et al., 2008).

Interestingly, annexin A2 (ANXA2) can bind the pseudoknot structure of avian coronavirus infectious bronchitis virus (IBV) and reduce −1 frameshifting (Kwak et al., 2011). As a consequence, ANXA2 has been suggested as a more general antiviral regulator in eukaryotic cells. Other potential anti-viral agents could either specifically bind to the frameshift signal (such as antisense oligonucleotides, non-coding RNAs, or frameshift signal binding compounds) or interfere with peptidyltransferase activity (e.g., anisomycin or sparsomycin) or eEF2 activity (e.g., sordarin) (Dinman, 2012b).

For certain diseases, however, it may be benefitial to enhance frameshifting. For instance, in monogenetic diseases such as cystic fibrosis or Duchenne's muscular dystrophy, where frameshift mutations result in premature translation termination, the deliberate induction of frameshifting may overcome the problem by skipping the affected sites (Aurino and Nigro, 2006). It has been noted that aminoglycosides such as gentamicin can enhance frameshifting and stop codon read-through. Gentamicin-induced read-through of stop codons has been evaluated as a treatment option for Duchenne's muscular dystrophy (Malik et al., 2010). While the authors conclude from this phase I clinical trial that gentamicin may not be a good treatment option, they note that other read-through agents may have benefits. A phase I/II clinical trial using a morpholino oligomer (AVI-4658) to correct a frameshift mutation in the *dystrophin* gene has been completed with the conclusion that AVI-4658 was well tolerated and had significant benefit in patients with Duchenne's muscular dystrophy (Cirak et al., 2011).

## Dual coding

Genome-wide analysis of the yeast genome (Jacobs et al., 2007) and other genomes (Hammell et al., 1999) suggests that frameshifting is more common than previously thought. However, it has been proposed that frameshifting may predominantly serve to modulate RNA levels rather than to produce frameshifted proteins (Plant et al., 2004). Evolutionary studies argue that the generation of out-of-frame proteins has been minimized by codon optimization that results in non-functional small peptides rather than functional proteins (Bollenbach et al., 2007). However, it is becoming increasingly recognized that programmed ribosomal frameshifting can result in peptides or proteins with physiological functions (Dinman, 2012a).

One question is whether frameshifting compromises the function of the original frame. It has been proposed that dual coding limits the evolutionary flexibility of the underlying nucleotide sequence (Firth and Brown, 2006; Rancurel et al., 2009). Thus, one possibility may be that frameshifting occurs predominantly in highly conserved, essential genes. However, it has been argued that once a frameshift event is released from selective pressure, as occurs in gene duplication, it can evolve to produce a beneficial functional protein (Raes and Van de Peer, 2005). Indeed, in some cases, novel genes seem to have emerged by frameshifting of a pre-existing coding sequence (Ohno, 1984; Ranz et al., 2003). Moreover, regulated frameshifting can allow the same gene to produce alternative beneficial proteins. In fact, evolutionary studies indicate a high abundance of frameshift events in human and mouse genomes that may be linked to an increased usage of the opal TGA stop codon (Okamura et al., 2006).

It has been proposed that at least 1% of the human genome consists of dual coding regions (Michel et al., 2012) and that the number of out-of-frame peptides or proteins may be even higher than that. Other studies have suggested that ~10% of the genome contains −1 frameshift signals (Belew et al., 2011). I would argue that the number of frameshifted peptides or proteins is somewhere in the range of 1–10% of the genome. This is a very significant fraction of the genome, thus suggesting that out-of-frame peptides are an inherent part of animal physiology and part of evolutionary selection processes. Accordingly, it can be anticipated that dual coding is regulated and a common mechanism for producing additional gene products. The main questions are:

1. What is the sequence or structural motif for dual coding?
2. What is the identity of all dual-coding genes and out-of-frame proteins in the genome?
3. How is dual coding regulated?

To answer these questions, we need to look at commonly used methods to identify frameshift events and out-of-frame peptides and proteins.

## Methods to identify out-of frame peptides

**Figure 3:**
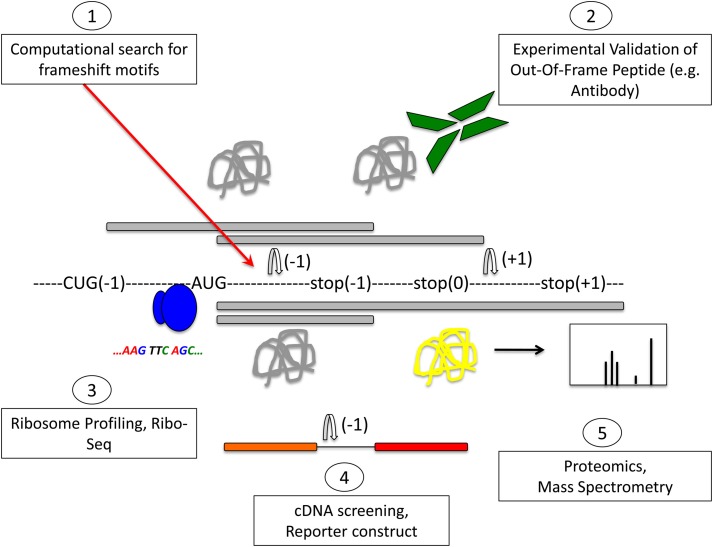
**Methods to detect frameshifting events and out-of-frame peptides.** Computational methods (1) using databases that interrogate the genome for −1 or +1 frameshift motifs can give information about frameshifting events. Limitations of this approach are that only known frameshift motifs are taken into account. Furthermore, this method does not give any information whether predicted frameshift events occur *in vivo* or the functional relevance of frameshifting events. Experimental methods (2) can identify frameshift events that occur *in vivo*. Most commonly, an antibody that is specific to the out-of-frame sequence of the frameshifted protein is used to detect frameshifted proteins. Ribosome profiling methods such as Ribo-Seq (3) can be used to detect out-of-frame peptides on a genomic scale. cDNA screening and the use of tandem luciferase reporter constructs (4) can be used to experimentally detect frameshift events. Limitations of this approach are that overexpression may result in dysregulated expression and differences in translation compared to endogenous expression levels. Proteomic methods (5) using mass spectrometry are suitable to detect endogenous out-of-frame peptides. However, the levels of frameshifted proteins may be low and escape the detection limit. A combination of these methods may provide most suitable to identify out-of-frame proteins on a genome-wide scale. See text for details.

Soon after the discovery of programmed ribosomal frameshifting, it was proposed that frameshifting may be a common mechanism for dual decoding of genetic information (Dinman, 2012a). The existence of an alternative genome has been postulated, but it has remained difficult to identify. Most methods aim to identify frameshifting events, but very few can determine which produce functional out-of-frame gene products. A summary of methods to identify frameshifting events in mRNAs and out-of-frame products is schematically shown in Figure 3.

Computational prediction has proven very useful. First, one has to identify in mRNAs the requirements for a productive frameshift peptide: One might argue that a slippery sequence and a pseudoknot are a good predictor of frameshifting events, but—as pointed out above—there are alternative mechanisms that result in frameshifting (see also Figure 2). For identification of the frameshifted products, I propose three hallmarks: first, the presence of an initiation triplet (AUG or CUG) and a stop codon in +1 or −1 frame; second, a stable peptide that follows the 50-nt rule (Nagy and Maquat, 1998; Hillman et al., 2004); and third, the validation that the peptide is endogenously expressed. Validation can be done by low-throughput experimental methods, either using antibodies raised against the frameshifted protein or using genetic manipulations that ablate the frameshift protein without disrupting the zero frame protein, if possible. To identify frameshifting on a genome-wide scale, higher throughput approaches can be applied, such as genomic, phenotypic, or proteomic screening.

### Computational prediction of frameshifting events

Several databases have been created to help predict frameshift sequences in the genome. Such predictions are based on primary mRNA sequence stretches or on secondary hairpins or pseudo-knots within the mRNA sequence. For instance, Hammell et al. searched the genome for slippery sequences and pseudoknot structures (Hammell et al., 1999). This approach identified over 200 putative programmed ribosomal frameshifting events. For identification of pseudoknots and slippery sequences in the genome databases such as RECODE, KnotInFrame, PRFdb, and FSdB are very useful (Table 1).

**Table 1:** **Databases for prediction of −1 and +1 frameshifting**.

**Database**	**Prediction motif**	**Pseudoknot**	**References**
RECODE	Known frameshift sequences (~1500 genes)	Pseudoviewer, ARFA, OAZ	http://recode.ucc.ie/
KnotInFrame	X XXY YYZ Spacer 1–12 nt	pKnotsRG-fs	http://bibiserv.techfak.uni-bielefeld.de/knotinframe/
PRFdb	X XXY YYZ Spacer 1–8 nt	RNAmotif, pKnots, Nupack, Hotknots	http://prfdb.umd.edu/
FSDB	See Table 2; 63 known frameshift sequences and 190 predicted sequences	FSFinder	http://wilab.inha.ac.kr/fsdb/

KnotInFrame and PRFdb rely entirely on computational prediction of a combination of a slippery sequence, a spacer and pseudoknot or stem-loop structure, while RECODE and FSDB compile known frameshift sequences in order to predict similar motifs in other genes.

RECODE provides information about programmed frameshifting, read-through and bypassing, based on published results in the literature (Baranov et al., 2001; Bekaert et al., 2010). The database utilizes information from ~1500 known frameshifted gene products. A majority of the data on frameshifting comes from two frameshifted proteins, RF2 and antizyme, and is constantly updated using the respective prediction tools [ARFA (Bekaert et al., 2006) and OAF (Bekaert et al., 2008), respectively]. PseudoViewer is used for visualization of pseudoknot structures. (http://recode.ucc.ie)

KnotInFrame is a program for predicting sites of −1 frameshifting based on the formation of RNA pseudoknots (Theis et al., 2008). The authors have developed a specialized RNA-folding program called pknotsRG-fs that compares the minimal free energy of an enforced pseudoknot structure to that of a freely folded structure such as that given by RNAfold. The spacer region after the common slippery sequence X XXY YYZ is between 1 and 12 nt long. (http://bibiserv.techfak.uni-bielefeld.de/knotinframe)

PRFdb (http://prfdb/umd.edu/) is limited to −1 frameshifting in eukaryotes only. Again, the prediction if based on the presence of a heptameric slippery sequence in combination with a pseudoknot (Jacobs et al., 2007). The slippery sequence is modeled with a 1–8 nt spacer, and the pseudoknot is identified using RNAMotif. The pseudoknot is then further confirmed with other secondary RNA-structure-prediction tools, including Pknots (Rivas and Eddy, 1999), Nupack (Dirks and Pierce, 2004), and HotKnots (Ren et al., 2005).

The Frameshift Signal Database (FSDB) is a compilation of all known frameshift motifs, plus some reported (predicted) frameshift sequences (Moon et al., 2007). Based on commonalities between these sequences, the associated FSFinder allows mining of the genome for potential frameshift sequences. (http://wilab.inha.ac.kr/fsdb) At present, the database contains a total of 63 experimental and 190 predicted sequences from viruses, prokaryotes and eukaryotes. FSFinder uses a combination of slippery sequences and pseudoknot or stem-loop prediction. The heptameric slippery sequences for +1 and −1 frameshifting are listed in Table 2. It is noteworthy that FSDB includes deviations from the standard X XXY YYZ slippery sequence for prediction of −1 frameshifting, whereas +1 frameshift sequences often contain stop codons.

**Table 2:** **Slippery sequences used by FSDB for prediction of out-of-frame peptides**.

**−1 Frameshift**	**+1 Frameshift**
AAAAAA C/G/U	AAA **UAA** A
AAAAG	CCC U
AAAUUU A/C/G/U	CCC **UGA**
CCCAAA A/C/G	CUU AGG
CCCUUU A	CUU **UAA** C
CGAAAG	CUU **UGA** C
GGAUUU A/U	GCG A
GGGAAA A/C/G/U	UCC **UGA**
GGGCCC C/U	UUU **UGA**
GGGGAA C	
GGGUUU A/C/U	
GUUAAA C	
UUUAAA A/C/U	
UUUUUU A/C/G	

Sequences predictive of −1 frameshifting include classical heptameric X XXY YYZ sequences and also include shorter sequence stretches. Sequences predictive of +1 frameshifting often contain an in-frame stop codon, thus resulting in stop codon by-passing such as in the case of ODC.

Although most prediction databases use similar principles (slippery sequence plus pseudoknot), they differ in prediction of frameshifting events. This may be due to variable thresholds applied or different RNA folding algorithms. In order to identify expressed proteins, one has to consider the length of the predicted out-of-frame protein. Most frameshifted proteins initiated by predicted slippery sites will terminate within 5–10 codons, thus producing truncated or non-functional peptides or proteins. Alternative approaches that take out-of-frame protein length into account might be beneficial. For instance, MLOGD (http://Guinevere.otago.ac.nz/aef/MLOGD) is a program for detection of overlapping coding sequences based on sequence alignments and analysis of mutation patterns (Firth and Brown, 2006). The limitations of this approach are that less conserved coding sequences will not be identified, and -2 frame overlaps can be identified as false positives.

In some organisms, including mammals, codon usage has evolved to minimize frameshifting. Thus, it can be expected that certain codons may favour frameshifting. FSCAN is a program to identify +1 frameshift sequences based on codon usage in *E. coli* (Liao et al., 2009). FScan searches 16 nt sequences and calculates a score for aa-tRNA competition between the zero and +1 frame. Accordingly, a stop codon, or a rare codon in the zero frame can be a predictor of +1 frameshifting. Shah et al. suggested that selective pressure would lead to an under-representation of frameshift sites in protein-coding sequences relative to an organism's codon bias (Shah et al., 2002). They predicted the sequences CUU AGG C and CUU AGU U, which mediate +1 frameshifting of ABP140 and EST3, respectively, to be highly under-represented and predictive for frameshifting in *S. cerevisiae*.

Once frameshifting events have been identified, it is important to characterize the gene products. One could apply the filters mentioned above, such as the 50-nt rule, although it is quite possible that short peptides are stable and endogenously expressed (Kondo et al., 2010; Ingolia et al., 2011). Ultimately, all predictions of frameshifted peptides need to be validated with experimental methods.

### Low-throughput experimental methods

Experimental methods include generation of frameshift-specific antibodies, reporter constructs, biophysical methods, and single molecule measurements. The most commonly used frameshift reporter is a tandem luciferase construct where the two luciferases with different substrate specificities are separated by a stretch of nucleotides of a length that shifts the downstream luciferase either one nucleotide up or down (Grentzmann et al., 1998). The downstream luciferase will be expressed only if the nucleotide stretch can mediate frameshifting, while the upstream luciferase serves as an expression control. Alternative reporter genes include the use of fluorescent proteins (Cardno et al., 2009), although the dynamic range of luciferases is generally much higher. This reporter can be used experimentally to confirm predicted frameshift events.

One hypothesis is that the −1 frameshifting efficiency correlates with the mechanical force required for pulling the RNA pseudoknot apart. With biophysical single-molecule methods that measure these forces using optical tweezers, Hansen et al. have confirmed that unfolding of a IBV-based pseudoknot required ~500 kJ/mol, compared to the theoretically determined 292 kJ/mol (Hansen et al., 2007). Chen et al. have determined that a 100% confidence in −1 frameshifting is reached by an unfolding force of ~57 pN (Chen et al., 2009). Such single-molecule biophysical approaches may help to identify potential frameshift sequences, but as they require immobilization of the RNA, they will be technically challenging to implement on a genome-wide scale.

An alternative is the use of single molecule Foerster Resonance Energy Transfer (smFRET) (Aitken and Puglisi, 2010). FRET is a useful technique to measure proximity of biomolecules. A donor fluorophore attached to a molecule can transfer photons to an acceptor fluorophore on a different molecule when both molecules are close together (generally less than 10 nm). Aitken and Puglisi have used this technique to label individual tRNA molecules with donor and acceptor dyes that result in energy transfer when in close proximity and correct orientation. This technique enables to monitor relative tRNA positions and movement of tRNAs on ribosomes at a millisecond scale. It has been used to identify ribosomal translocation events on fluorescent ribosomes on immobilized RNA sequences and predicted the slipperiness of various RNA sequences. Again, this will be technically difficult to implement on a genome-wide scale.

**Figure 4:**
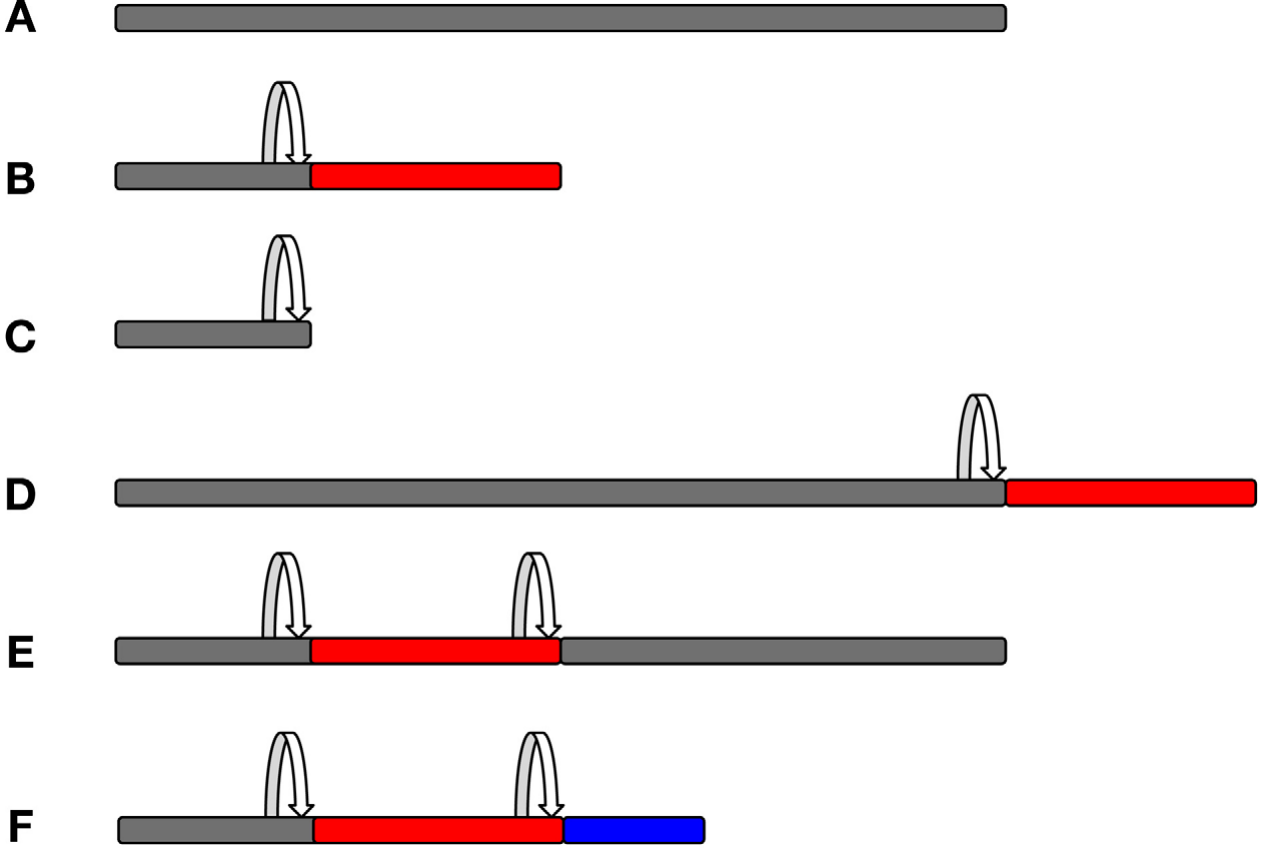
**Protein composition as a consequence of frameshifting.** The original zero frame is shown in gray, whereas out-of-frame sequences are shown in red or blue colour. A frameshifting event is marked by the arrow. Frameshifting can result in chimeric peptides composed of the original frame and out-of-frame sequences that can form a small or larger part of the overall protein, depending on where the frameshift event takes place. It is also possible that more than one frameshifting event takes place within the same mRNA, thus resulting in mosaic hybrid peptide sequences. (**A**, Original frame; **B**, early frameshift with extended ORF; **C**, early frameshift with truncation; **D**, late frameshift with extended ORF; **E**, two frameshift events that switch back to the zero frame; and **F**, two frameshift events that produce a chimeric sequence of three different frames.)

All of the above methods will record mostly frameshifting events, but they will not validate the expression of an out-of-frame protein. It is worth noting that frameshifting will most likely result in chimeric sequences composed of a stretch of zero frame peptides linked to out-of-frame peptides, which can be a small or large part of the overall protein, depending on where the frameshift site is. Alternatively, frameshifting could lead to truncation of the original protein, where a very small fraction—if any—of the overall protein is out-of-frame (Figure 4). Those proteins that have a sufficient predicted length could then be validated by raising specific antibodies against that sequence. In order to raise an antibody, one has to know the precise sequence of the frameshifted protein. Therefore, this approach is useful for confirming a known out-of-frame protein, but it is not amenable to large-scale genome-wide screening. In addition, the detection of an endogenously expressed out-of-frame protein does not necessarily indicate a functional relevance for this protein.

In order to validate a physiological role for the frameshifted gene product, the best method is to genetically ablate the expression of the frameshifted protein while preserving the in-frame sequence. This can be accomplished by gene targeting with a gene sequence that harbours multiple wobble base pair mutations or mutating the frameshift motif, thus altering the RNA sequence but not the in-frame protein sequence. This is technically challenging and may not always be feasible. For instance, an OAZ3 knockout mouse model has been generated, where both the zero frame and the +1 frame has been deleted (Tokuhiro et al., 2009). To my knowledge, no specific gene targeting of a frameshift protein has been done so far.

### Genomic methods

Genomic methods such as RNA sequencing (RNA-seq) have emerged as powerful tools to profile RNA content in cells. RNA-seq is based on high-throughput sequencing of a cDNA library generated from cellular RNA. In its standard form, RNA-seq enables the identification of indels, but will fail to identify post-transcriptional phenotypes and will therefore fail to identify programmed ribosomal frameshifting or dual coded genes. Nevertheless, ribosome profiling methods such as Ribo-seq have been developed that allow the identification of active translation, based on sequencing of cDNA libraries generated from ribosome-protected fragments (Ingolia et al., 2009). In this case, mRNA bound to ribosomes is first cross-linked and then isolated using, e.g., sucrose gradient density centrifugation. Next, a nuclease digestion step results in removal of mRNA sequence that is not bound (“protected”) to ribosomes. The protected RNA bound to ribosomes is then reverse transcribed into cDNA and sequenced. Therefore, the precise position of a ribosome can be matched to the site of active translation. An adaptation of this method using the drug harringtonine to cause ribosome accumulation at initiation codons has allowed the identification of translation start sites and confirmed that many proteins are initiated at non-AUG or alternative AUG sites (Ingolia et al., 2011). In this study, 44% of detected AUG start sites were unannotated, and a large fraction of these encoded out-of-frame peptides. Further uses of ribosome profiling have confirmed that the identification of frameshifts is possible using genomic technologies (Michel et al., 2012). Although this approach is unbiased in the sense that it does not pre-filter genetic regions, one problem is the non-uniformity of ribosome-protected fragment reads. For instance, the preparation of cDNA libraries generated from ribosome-protected fragments can result in over- or under-representation of sequence reads. This can be overcome by a computational approach that measures the cumulative subcodon proportion difference of ribosome-protected fragments relative to local subcodon positions. The authors have therefore combined experimental data from Ribo-Seq with a computational approach in order to identify novel frameshifted protein sequences. Using this approach, several new frameshifted protein sequences were identified, most of which were dual-encoded. The authors estimate that more than 1% of the genome may consist of dual-coding regions, and this is likely an underestimation. Further improvements to the method, including deeper sequencing to get better coverage of ribosome-protected fragments, will help to identify these genes.

### Phenotypic screening

Most genome-wide screening approaches such as RNAi-based knockdown methods will fail to identify dual-coding regions, as both gene products will be deleted. Further, most commonly used cDNA libraries such as the ORFeome are designed to avoid expression of frameshifted peptides—for instance, by deletion of the 5′ and 3′-UTR. It may, however, be possible that certain phenotypes in cDNA screening are exerted by out-of-frame proteins. In order to fish for phenotypic effects exerted by such peptides, cDNA libraries can be designed in a way that genomic fragments are inserted downstream of an AUG start codon in +1 or −1 frame so that all genomic fragments are deliberately frameshifted. A subsequent functional phenotypic screen can then identify phenotypes associated with expression of such deliberate frameshift fragments. However, there are limitations in such an approach, e.g., where to place the frameshift. Also, deliberately expressed out-of-frame proteins may have little physiological relevance.

An alternative may be to use agents such as gentamicin that enhance programmed frameshifting. In that case, cells expressing a cDNA library would be treated with a frameshift inducer, and the occurrence of differential phenotypes plus/minus frameshift inducer would be recorded. It is imperative that cDNA libraries with 3′-UTR regions are used in such an approach to facilitate the out-of-frame peptide expression after stop codon read-through.

A similar approach can be used to identify endogenous regulators of frameshifting. One could use the dual luciferase reporter construct (Grentzmann et al., 1998) with a known frameshift motif and screen siRNA or cDNA libraries to identify genes that enhance or inhibit the frameshifting efficiency of this reporter. The existence of non-coding RNAs that modulate frameshifting suggests that frameshifting is regulated by endogenous gene products.

The dual luciferase reporter mentioned above can also be used to probe the sequence space for optimal frameshifting motifs (Rakauskaite et al., 2011). Recently, an adaptation of this approach has been developed using fluorophores that are amenable to high-throughput screening applications (Cardno et al., 2009). An *in vivo* adaptation for yeast cells has been proposed for use in high-throughput screening experiments (Harger and Dinman, 2003). For instance, a random nucleotide sequence can be inserted between the in-frame and the out-of-frame luciferase to determine which sequence will result in high levels of out-of-frame expression. The main advantages of such a system are the broad linear range of the assay, the internal mRNA expression control (luciferase 1), the possibility to normalize relative frameshift expression, and the ease of use. One problem with the approach is that the secondary structure is affected by the length of the sequence inserted in the dual luciferase reporter. Thus, the context of the frameshift motifs needs to be taken into account. Further, the high number of potential nucleotide combinations may outweigh the capacity of even highly automated processes. Even though this will identify putative sequences with high potential for frameshifting, it is still not clear whether the out-of-frame peptides or proteins are stable and functional. Their presence in open reading frames may hint that a frameshift is buried within the gene. Subsequent low-throughput experimental methods (see above) need to be designed to confirm expression of the frameshifted protein.

### Proteomics

Proteomic approaches have enabled the identification of expressed peptides under physiological conditions. In mass spectrometry (MS), peptides of endogenous proteins are first detected as a mass per ion. In the most common approach, recorded masses are matched to all potential masses in the respective database by search algorithms like SEQUEST, MASCOT, Andromeda, or PEAKS. In a next step, sequence information generated in tandem MS (MS/MS) experiments is employed to identify potential hits within the shortlisted peptide variants. Commonly searched databases by MASCOT are SwissProt, NCBInr, and EMBL EST. These databases integrate cDNA and EST sequences and generally do not contain out-of-frame peptides. However, as MS database search algorithms compute the entire sequence space of potential peptide matches to identify peptides within analyzed samples, it is in principle possible to identify out-of-frame peptides.

An interesting approach for the identification of frame-shift peptides is *de novo* sequence analysis by MS. This is based solely on the analysis of MS and MS/MS spectra, without amino acid sequence information from databases. However, identification rates are typically lower than in classical database-based proteomics experiments.

A major difficulty is to assign frame-shifted peptides to a particular gene. Decoding of the underlying nucleotide sequence is sometimes problematic, as, for instance, isoleucine and leucine have the same exact mass. In some cases, the identified peptides do not match the database for various reasons, including errors in gene/protein annotation and post-translational modifications in the peptides that are not accounted for. Usually, unmatched peptides are not reported in publications and are disregarded from further analysis. It is possible that some of these unmatched peptides correspond to an alternative reading frame. In order to match these peptides, one would need to generate a database derived from the +1/−1 frames of the ORFeome, similar to what Okamura et al. have done (Okamura et al., 2006), and make these accessible to the proteomic community. One problem is the “breakpoint” of frameshifting, which would generate a peptide that is partially composed of the original frame and partially of the frameshifted peptide (see Figure 4). Another problem in this approach may be the paucity of frameshifted peptides, as they are commonly expressed at lower levels (see above) and may not be easily detectable by MS approaches. However, MS is probably the most powerful method to identify out-of-frame peptides to confirm endogenous expression.

An alternative is to study peptides presented by cell-surface, class I MHC proteins. They present peptides derived from intracellular proteins to enable immune tolerance and immune surveillance. MHC-presented out-of-frame peptides were discovered in the early 1990's (Shastri et al., 1995) and have enabled the unbiased identification of endogenous frameshifting in mammalian cells long before the technical advances of MS. However, it is technically challenging to use this approach as a systematic tool for the identification of genomic out-of-frame peptides.

## Outlook

In which cellular processes should we expect to see a high abundance of frameshifted proteins? In principle, such proteins might be involved in any cellular process, but may be correlated with certain cellular pathways. For instance, amino acid starvation can induce frameshifting in bacteria (Barak et al., 1996). One hypothesis is that this is due to a short supply of amino-acylated tRNA as a consequence of amino acid limitations. This is supported by the observation that antizyme expression (the +1 frame) is maintained under conditions of amino acid starvation, while expression of ODC (the 0 frame) is reduced in rat intestinal epithelial cells (Ray et al., 2012). Moreover, treatment with mTOR inhibitors such as rapamycin also reduce 0 frame expression while maintaining antizyme expression. One could hypothesize that reduced fidelity in translation may enhance frameshifting. On the other hand, both translation inhibition and amino acid starvation are conditions that increase autophagy in eukaryotic cells, raising the possibility that the production of out-of-frame proteins may be functionally coupled to the regulation of autophagy.

## Conclusion

There is increasing evidence for an alternative genome/proteome in both prokaryotes and eukaryotes, reflecting programmed ribosomal frameshifting. It is likely that a combination of computational, experimental, genomic, and proteomic methods will be needed to determine the entire frameshifted proteome, as required to understand fully gene expression in any organism. We need to identify the frameshift motifs that enable frameshifting, as well as all the genes that produce out-of-frame peptides. Finally, as a low level of frameshifting can be considered “biological noise,” we need to determine the physiological relevance of these frameshifted proteins.

### Conflict of interest statement

The author declares that the research was conducted in the absence of any commercial or financial relationships that could be construed as a potential conflict of interest.

## References

[B1] Aitken C. E. Puglisi J. D. (2010). Following the intersubunit conformation of the ribosome during translation in real time. Nat. Struct. Mol. Biol. 17, 793–800 10.1038/nsmb.1828 20562856 PMC4459212

[B2] Aurino S. Nigro V. (2006). Readthrough strategies for stop codons in Duchenne muscular dystrophy. Acta myol. 25, 5–12 17039975

[B3] Barak Z. Lindsley D. Gallant J. (1996). On the mechanism of leftward frameshifting at several hungry codons. J. Mol. Biol. 256, 676–684 10.1006/jmbi.1996.0117 8642590

[B4] Baranov P. V. Gurvich O. L. Fayet O. Prere M. F. Miller W. A. Gesteland R. F. (2001). RECODE: a database of frameshifting, bypassing and codon redefinition utilized for gene expression. Nucleic Acids Res. 29, 264–267 10.1093/nar/29.1.264 11125107 PMC29850

[B5] Baranov P. V. Wills N. M. Barriscale K. A. Firth A. E. Jud M. C. Letsou A. (2011). Programmed ribosomal frameshifting in the expression of the regulator of intestinal stem cell proliferation, adenomatous polyposis coli (APC). RNA Biol. 8, 637–647 10.4161/rna.8.4.15395 21593603 PMC3225980

[B6] Bekaert M. Atkins J. F. Baranov P. V. (2006). ARFA: a program for annotating bacterial release factor genes, including prediction of programmed ribosomal frameshifting. Bioinformatics 22, 2463–2465 10.1093/bioinformatics/btl430 16895933

[B7] Bekaert M. Firth A. E. Zhang Y. Gladyshev V. N. Atkins J. F. Baranov P. V. (2010). Recode-2: new design, new search tools, and many more genes. Nucleic Acids Res. 38, D69–D74 10.1093/nar/gkp788 19783826 PMC2808893

[B8] Bekaert M. Ivanov I. P. Atkins J. F. Baranov P. V. (2008). Ornithine decarboxylase antizyme finder (OAF): fast and reliable detection of antizymes with frameshifts in mRNAs. BMC Bioinformatics 9:178 10.1186/1471-2105-9-178 18384676 PMC2375905

[B9] Belew A. T. Advani V. M. Dinman J. D. (2011). Endogenous ribosomal frameshift signals operate as mRNA destabilizing elements through at least two molecular pathways in yeast. Nucleic Acids Res. 39, 2799–2808 10.1093/nar/gkq1220 21109528 PMC3074144

[B10] Bollenbach T. Vetsigian K. Kishony R. (2007). Evolution and multilevel optimization of the genetic code. Genome Res. 17, 401–404 10.1101/gr.6144007 17351130

[B11] Brakier-Gingras L. Charbonneau J. Butcher S. E. (2012). Targeting frameshifting in the human immunodeficiency virus. Expert Opin. Ther. Targets 16, 249–258 10.1517/14728222.2012.665879 22404160 PMC3328590

[B13] Brierley I. Dos Ramos F. J. (2006). Programmed ribosomal frameshifting in HIV-1 and the SARS-CoV. Virus Res. 119, 29–42 10.1016/j.virusres.2005.10.008 16310880 PMC7114087

[B14] Cardno T. S. Poole E. S. Mathew S. F. Graves R. Tate W. P. (2009). A homogeneous cell-based bicistronic fluorescence assay for high-throughput identification of drugs that perturb viral gene recoding and read-through of nonsense stop codons. RNA 15, 1614–1621 10.1261/rna.1586709 19535460 PMC2714747

[B15] Chen G. Chang K.-Y. Chou M.-Y. Bustamante C. Tinoco I. Jr. (2009). Triplex structures in an RNA pseudoknot enhance mechanical stability and increase efficiency of −1 ribosomal frameshifting. Proc. Natl. Acad. Sci. U.S.A. 106, 12706–12711 10.1073/pnas.0905046106 19628688 PMC2722267

[B16] Chen X. Kang H. Shen L. X. Chamorro M. Varmus H. E. Tinoco I. Jr. (1996). A characteristic bent conformation of RNA pseudoknots promotes −1 frameshifting during translation of retroviral RNA. J. Mol. Biol. 260, 479–483 10.1006/jmbi.1996.0415 8759314

[B17] Cirak S. Arechavala-Gomeza V. Guglieri M. Feng L. Torelli S. Anthony K. (2011). Exon skipping and dystrophin restoration in patients with Duchenne muscular dystrophy after systemic phosphorodiamidate morpholino oligomer treatment: an open-label, phase 2, dose-escalation study. Lancet 378, 595–605 10.1016/S0140-6736(11)60756-3 21784508 PMC3156980

[B18] Clark M. B. Janicke M. Gottesbuhren U. Kleffmann T. Legge M. Poole E. S. (2007). Mammalian gene PEG10 expresses two reading frames by high efficiency −1 frameshifting in embryonic-associated tissues. J. Biol. Chem. 282, 37359–37369 10.1074/jbc.M705676200 17942406

[B19] Dinman J. D. (2012a). Control of gene expression by translational recoding. Adv. Protein Chem. Struct. Biol. 86, 129–149 10.1016/B978-0-12-386497-0.00004-9 22243583 PMC7149833

[B20] Dinman J. D. (2012b). Mechanisms and implications of programmed translational frameshifting. Wiley Interdiscip. Rev. RNA 3, 661–673 10.1002/wrna.1126 22715123 PMC3419312

[B22] Dirks R. M. Pierce N. A. (2004). An algorithm for computing nucleic acid base-pairing probabilities including pseudoknots. J. Comp. Chem. 25, 1295–1304 10.1002/jcc.20057 15139042

[B23] Dulude D. Berchiche Y. A. Gendron K. Brakier-Gingras L. Heveker N. (2006). Decreasing the frameshift efficiency translates into an equivalent reduction of the replication of the human immunodeficiency virus type 1. Virology 345, 127–136 10.1016/j.virol.2005.08.048 16256163

[B24] Firth A. E. Brown C. M. (2006). Detecting overlapping coding sequences in virus genomes. BMC Bioinformatics 7:75 10.1186/1471-2105-7-75 16483358 PMC1395342

[B25] Gendron K. Charbonneau J. Dulude D. Heveker N. Ferbeyre G. Brakier-Gingras L. (2008). The presence of the TAR RNA structure alters the programmed −1 ribosomal frameshift efficiency of the human immunodeficiency virus type 1 (HIV-1) by modifying the rate of translation initiation. Nucleic Acids Res. 36, 30–40 10.1093/nar/gkm906 17984074 PMC2248755

[B26] Grentzmann G. Ingram J. A. Kelly P. J. Gesteland R. F. Atkins J. F. (1998). A dual-luciferase reporter system for studying recoding signals. RNA 4, 479–486 9630253 PMC1369633

[B27] Gurvich O. L. Baranov P. V. Gesteland R. F. Atkins J. F. (2005). Expression levels influence ribosomal frameshifting at the tandem rare arginine codons AGG_AGG and AGA_AGA in *Escherichia coli*. J. Bacteriol. 187, 4023–4032 10.1128/JB.187.12.4023-4032.2005 15937165 PMC1151738

[B28] Hammell A. B. Taylor R. C. Peltz S. W. Dinman J. D. (1999). Identification of putative programmed −1 ribosomal frameshift signals in large DNA databases. Genome Res. 9, 417–427 10.1101/gr.9.5.417 10330121 PMC310776

[B29] Hansen T. M. Reihani S. N. S. Oddershede L. B. Sorensen M. A. (2007). Correlation between mechanical strength of messenger RNA pseudoknots and ribosomal frameshifting. Proc. Natl. Acad. Sci. U.S.A. 104, 5830–5835 10.1073/pnas.0608668104 17389398 PMC1838403

[B30] Harger J. W. Dinman J. D. (2003). An *in vivo* dual-luciferase assay system for studying translational recoding in the yeast *Saccharomyces cerevisiae*. RNA 9, 1019–1024 10.1261/rna.5930803 12869712 PMC1236998

[B31] Hiller M. Huse K. Platzer M. Backofen R. (2005). Creation and disruption of protein features by alternative splicing – a novel mechanism to modulate function. Genome Biol. 6, R58 10.1186/gb-2005-6-7-r58 15998447 PMC1175989

[B32] Hillman R. T. Green R. E. Brenner S. E. (2004). An unappreciated role for RNA surveillance. Genome Biol. 5, R8 10.1186/gb-2004-5-2-r8 14759258 PMC395752

[B33] Ingolia N. T. Ghaemmaghami S. Newman J. R. S. Weissman J. S. (2009). Genome-wide analysis *in vivo* of translation with nucleotide resolution using ribosome profiling. Science 324, 218–223 10.1126/science.1168978 19213877 PMC2746483

[B34] Ingolia N. T. Lareau L. F. Weissman J. S. (2011). Ribosome profiling of mouse embryonic stem cells reveals the complexity and dynamics of mammalian proteomes. Cell 147, 789–802 10.1016/j.cell.2011.10.002 22056041 PMC3225288

[B35] Ivanov I. P. Gesteland R. F. Atkins J. F. (2000a). Antizyme expression: a subversion of triplet decoding, which is remarkably conserved by evolution, is a sensor for an autoregulatory circuit. Nucleic Acids Res. 28, 3185–3196 10.1093/nar/28.17.3185 10954585 PMC110703

[B36] Ivanov I. P. Matsufuji S. Murakami Y. Gesteland R. F. Atkins J. F. (2000b). Conservation of polyamine regulation by translational frameshifting from yeast to mammals. EMBO J. 19, 1907–1917 10.1093/emboj/19.8.1907 10775274 PMC302018

[B37] Jacobs J. L. Belew A. T. Rakauskaite R. Dinman J. D. (2007). Identification of functional, endogenous programmed −1 ribosomal frameshift signals in the genome of *Saccharomyces cerevisiae*. Nucleic Acids Res. 35, 165–174 10.1093/nar/gkl1033 17158156 PMC1802563

[B38] Kobayashi Y. Zhuang J. Peltz S. Dougherty J. (2010). Identification of a cellular factor that modulates HIV-1 programmed ribosomal frameshifting. J. Biol. Chem. 285, 19776–19784 10.1074/jbc.M109.085621 20418372 PMC2888388

[B39] Kondo T. Plaza S. Zanet J. Benrabah E. Valenti P. Hashimoto Y. (2010). Small peptides switch the transcriptional activity of Shavenbaby during Drosophila embryogenesis. Science 329, 336–339 10.1126/science.1188158 20647469

[B40] Kwak H. Park M. W. Jeong S. (2011). Annexin A2 binds RNA and reduces the frameshifting efficiency of infectious bronchitis virus. PLoS ONE 6:e24067 10.1371/journal.pone.0024067 21918681 PMC3168876

[B41] Laine S. Thouard A. Komar A. A. Rossignol J.-M. (2008). Ribosome can resume the translation in both +1 or −1 frames after encountering an AGA cluster in *Escherichia coli*. Gene 412, 95–101 10.1016/j.gene.2008.01.018 18313865

[B42] Liao P.-Y. Choi Y. S. Lee K. H. (2009). FSscan: a mechanism-based program to identify +1 ribosomal frameshift hotspots. Nucleic Acids Res. 37, 7302–7311 10.1093/nar/gkp796 19783813 PMC2790909

[B43] Maia I. G. Seron K. Haenni A. L. Bernardi F. (1996). Gene expression from viral RNA genomes. Plant Mol. Biol. 32, 367–391 8980488 10.1007/BF00039391

[B44] Malik V. Rodino-Klapac L. R. Viollet L. Wall C. King W. Al-Dahhak R. (2010). Gentamicin-induced readthrough of stop codons in Duchenne muscular dystrophy. Ann. Neurol. 67, 771–780 10.1002/ana.22024 20517938

[B45] Martin R. Mogg A. E. Heywood L. A. Nitschke L. Burke J. F. (1989). Aminoglycoside suppression at UAG, UAA and UGA codons in *Escherichia coli* and human tissue culture cells. Mol. Gen. Genet. 217, 411–418 2475756 10.1007/BF02464911

[B46] Masuda I. Matsuzaki M. Kita K. (2010). Extensive frameshift at all AGG and CCC codons in the mitochondrial cytochrome c oxidase subunit 1 gene of *Perkinsus marinus* (Alveolata; Dinoflagellata). Nucleic Acids Res. 38, 6186–6194 10.1093/nar/gkq449 20507907 PMC2952869

[B47] Michel A. M. Choudhury K. R. Firth A. E. Ingolia N. T. Atkins J. F. Baranov P. V. (2012). Observation of dually decoded regions of the human genome using ribosome profiling data. Genome Res. [Epub ahead of print]. 10.1101/gr.133249.111 22593554 PMC3483551

[B48] Moon S. Byun Y. Han K. (2007). FSDB: a frameshift signal database. Comp. Biol. Chem. 31, 298–302 10.1016/j.compbiolchem.2007.05.004 17631420 PMC7185369

[B49] Murakami Y. Tanaka K. Matsufuji S. Miyazaki Y. Hayashi S. (1992). Antizyme, a protein induced by polyamines, accelerates the degradation of ornithine decarboxylase in Chinese-hamster ovary-cell extracts. Biochem. J. 283(Pt 3), 661–664 1590755 10.1042/bj2830661 PMC1130936

[B50] Nagy E. Maquat L. E. (1998). A rule for termination-codon position within intron-containing genes: when nonsense affects RNA abundance. Trends Biochem. Sci. 23, 198–199 9644970 10.1016/s0968-0004(98)01208-0

[B51] Namy O. Moran S. J. Stuart D. I. Gilbert R. J. Brierley I. (2006). A mechanical explanation of RNA pseudoknot function in programmed ribosomal frameshifting. Nature 441, 244–247 10.1038/nature04735 16688178 PMC7094908

[B52] Namy O. Rousset J. P. Napthine S. Brierley I. (2004). Reprogrammed genetic decoding in cellular gene expression. Mol. Cell 13, 157–168 10.1016/S1097-2765(04)00031-0 14759362

[B53] Nilsson J. Koskiniemi S. Persson K. Grahn B. Holm I. (1997). Polyamines regulate both transcription and translation of the gene encoding ornithine decarboxylase antizyme in mouse. Eur. J. Biochem. 250, 223–231 10.1111/j.1432-1033.1997.0223a.x 9428668

[B54] Ohno S. (1984). Birth of a unique enzyme from an alternative reading frame of the preexisted, internally repetitious coding sequence. Proc. Natl. Acad. Sci. U.S.A. 81, 2421–2425 6585807 10.1073/pnas.81.8.2421 PMC345072

[B55] Okamura K. Feuk L. Marques-Bonet T. Navarro A. Scherer S. W. (2006). Frequent appearance of novel protein-coding sequences by frameshift translation. Genomics 88, 690–697 10.1016/j.ygeno.2006.06.009 16890400

[B57] Plant E. P. Wang P. Jacobs J. L. Dinman J. D. (2004). A programmed −1 ribosomal frameshift signal can function as a cis-acting mRNA destabilizing element. Nucleic Acids Res. 32, 784–790 10.1093/nar/gkh256 14762205 PMC373365

[B58] Raes J. Van de Peer Y. (2005). Functional divergence of proteins through frameshift mutations. Trends Genet. 21, 428–431 10.1016/j.tig.2005.05.013 15951050

[B59] Rakauskaite R. Liao P.-Y. Rhodin M. H. J. Lee K. Dinman J. D. (2011). A rapid, inexpensive yeast-based dual-fluorescence assay of programmed–1 ribosomal frameshifting for high-throughput screening. Nucleic Acids Res. 39, e97 10.1093/nar/gkr382 21602263 PMC3152369

[B60] Rancurel C. Khosravi M. Dunker A. K. Romero P. R. Karlin D. (2009). Overlapping genes produce proteins with unusual sequence properties and offer insight into *de novo* protein creation. J. Virol. 83, 10719–10736 10.1128/JVI.00595-09 19640978 PMC2753099

[B61] Ranz J. M. Ponce A. R. Hartl D. L. Nurminsky D. (2003). Origin and evolution of a new gene expressed in the Drosophila sperm axoneme. Genetica 118, 233–244 12868612

[B62] Ray R. M. Viar M. J. Johnson L. R. (2012). Amino acids regulate expression of antizyme-1 to modulate ornithine decarboxylase activity. J. Biol. Chem. 287, 3674–3690 10.1074/jbc.M111.232561 22157018 PMC3281678

[B63] Ren J. Rastegari B. Condon A. Hoos H. H. (2005). HotKnots: heuristic prediction of RNA secondary structures including pseudoknots. RNA 11, 1494–1504 10.1261/rna.7284905 16199760 PMC1370833

[B64] Rivas E. Eddy S. R. (1999). A dynamic programming algorithm for RNA structure prediction including pseudoknots. J. Mol. Biol. 285, 2053–2068 10.1006/jmbi.1998.2436 9925784

[B65] Seligmann H. (2010). An overlapping genetic code for frameshifted overlapping genes in Drosophila mitochondria: antisense antitermination tRNAs UAR insert serine. J. Theor. Biol. 298, 51–76 10.1016/j.jtbi.2011.12.026 22244915

[B66] Shah A. A. Giddings M. C. Parvaz J. B. Gesteland R. F. Atkins J. F. Ivanov I. P. (2002). Computational identification of putative programmed translational frameshift sites. Bioinformatics 18, 1046–1053 10.1093/bioinformatics/18.8.1046 12176827

[B67] Shastri N. Nguyen V. Gonzalez F. (1995). Major histocompatibility class I molecules can present cryptic translation products to T-cells. J. Biol. Chem. 270, 1088–1091 10.1074/jbc.270.3.1088 7836364

[B68] Temperley R. Richter R. Dennerlein S. Lightowlers R. N. Chrzanowska-Lightowlers Z. M. (2010). Hungry codons promote frameshifting in human mitochondrial ribosomes. Science 327, 301 10.1126/science.1180674 20075246

[B69] Theis C. Reeder J. Giegerich R. (2008). KnotInFrame: prediction of −1 ribosomal frameshift events. Nucleic Acids Res. 36, 6013–6020 10.1093/nar/gkn578 18820303 PMC2566878

[B70] Tholstrup J. Oddershede L. B. Sorensen M. A. (2011). mRNA pseudoknot structures can act as ribosomal roadblocks. Nucleic Acids Res. 40, 303–313 10.1093/nar/gkr686 21908395 PMC3245918

[B71] Tokuhiro K. Isotani A. Yokota S. Yano Y. Oshio S. Hirose M. (2009). OAZ-t/OAZ3 is essential for rigid connection of sperm tails to heads in mouse. PLoS Genet. 5:e1000712 10.1371/journal.pgen.1000712 19893612 PMC2763286

[B72] Toulouse A. Au-Yeung F. Gaspar C. Roussel J. Dion P. Rouleau G. A. (2005). Ribosomal frameshifting on MJD-1 transcripts with long CAG tracts. Hum. Mol. Genet. 14, 2649–2660 10.1093/hmg/ddi299 16087686

